# 12-week combined strength and endurance exercise attenuates CD8^+^ T-cell differentiation and affects the kynurenine pathway in the elderly: a randomized controlled trial

**DOI:** 10.1186/s12979-023-00347-7

**Published:** 2023-05-09

**Authors:** Tim Konstantin Boßlau, Paulina Wasserfurth, Thomas Reichel, Christopher Weyh, Jana Palmowski, Josefine Nebl, Niklas Joisten, Sergen Belen, Alexander Schenk, Andreas Hahn, Philipp Zimmer, Karsten Krüger

**Affiliations:** 1 Department of Exercise Physiology and Sports Therapy, Institute of Sports Science, Justus-Liebig-University Giessen, Kugelberg 62, 35394 Giessen, Germany; 2 Department of Exercise, Nutrition and Health, Faculty of Sport and Health Sciences, Technical University Munich, Connollystraße 32, 80809 Munich, Germany; 3 Faculty of Natural Sciences, Institute of Food Science and Human Nutrition, Leibniz University Hanover, Am Kleinen Felde 30, 30159 Hannover, Germany; 4 Division of Performance and Health, Institute for Sport and Sport Science, Technical University Dortmund, Otto-Hahn-Str. 3, 44227 Dortmund, Germany

**Keywords:** Ageing, CD8^+^ EMRA T-Cells, Kynurenine Pathway, Exercise, Nutrition, Insulin Resistance

## Abstract

**Background:**

Age-related accumulation of highly differentiated CD8^+^ effector memory re-expressing CD45RA (EMRA) T-cells and disruption of the kynurenine (KYN) pathway are associated with chronic inflammation and the development of insulin resistance.

In this study the aim was to investigate the effects of 12-week combined strength and endurance exercise on CD8^+^ T-cell differentiation and KYN pathway metabolites. Ninety-six elderly subjects (f/m, aged 50—70) were randomized to a control (CON) or exercise (EX) group. The EX group completed combined strength and endurance training twice weekly for one hour each time at an intensity of 60% of the one-repetition maximum for strength exercises and a perceived exertion of 15/20 for endurance exercises. The EX group was also randomly subdivided into two groups with or without a concomitant balanced diet intervention in order to examine additional effects besides exercise alone. Before and after the intervention phase, the proportions of CD8^+^ T-cell subsets and levels of KYN pathway metabolites in peripheral blood were determined.

**Results:**

The CD8^+^ EMRA T-cell subsets increased in the CON group but remained almost unchanged in the EX group (*p* = .02). Plasma levels of kynurenic acid (KA) increased in the EX group and decreased in the CON group (*p* = .03). Concomitant nutritional intervention resulted in lower levels of quinolinic acid (QA) compared with exercise alone (*p* = .03). Overall, there was a slight increase in the QA/KA ratio in the CON group, whereas it decreased in the EX group (*p* > .05).

**Conclusions:**

Combined strength and endurance training seems to be a suitable approach to attenuate CD8^+^ T-cell differentiation in the elderly and to redirect the KYN pathway towards KA. The clinical relevance of these effects needs further investigation.

**Supplementary Information:**

The online version contains supplementary material available at 10.1186/s12979-023-00347-7.

## Background

Ageing and persistent viral infections lead to changes within the peripheral CD8^+^ T-cell compartment. The proportion of naïve cells decreases, reducing the body’s ability to target new antigens, resulting in enhanced susceptibility to infection and diminished vaccine efficacy [1]. In parallel, constant cell stimulation leads to an increased proportion of highly differentiated cells, which can be classified as effector memory T-cells re-expressing CD45RA (EMRA T-cells) based on their CCR7^−^/CD45RA^+^ surface expression profile [2–4]. The chemokine receptor CCR7, which enables migration of naïve and central memory T-cells into lymph nodes, is lost in EMRA T-cells [5]. In contrast, CD45RA, an isoform of the common leukocyte antigen found on the surface of naïve T cells and which is also lost after activation, is re-expressed at highly differentiated developmental stages [6]. CD8^+^ EMRA T-cells are a heterogeneous composition of subsets associated with cell senescence, as they have reduced proliferative capacity [7], exhibit increased activation of senescence signalling pathways [8] and are associated with senescence markers such as KLRG1 and CD57 [4]. However, CD8^+^ EMRA T-cells are not functionally inactive, as they can migrate into inflamed tissues [9, 10] and produce a substantial amount of pro-inflammatory cytokines, like the senescence-associated secretory phenotype (SASP) seen in non-immune cells [11]. Thus, accelerated T-cell differentiation is possibly related to the development of chronic low-grade inflammation. This could be of high clinical relevance, as “Inflamm-aging” plays a considerable role in the development of age-related multimorbidity, including cancer, dementia, osteoporosis, and metabolic diseases [12].

Interestingly, the main degradation pathway of tryptophan (TRP), the kynurenine (KYN) pathway, interacts with the immune system. On the one hand, local and systemic inflammatory stimuli increase the expression of the initial and rate-limiting enzyme indoleamine 2–3-dioxygenase (IDO) in various tissues, such as peripheral blood mononuclear cells (PBMCs) [13, 14]. On the other hand, both KYN and one of its metabolites, kynurenic acid (KA), have immunomodulatory effects themselves and can activate the aryl hydrocarbon receptor (AHR), which plays a key role in T-cell differentiation and the induction of inflammatory mediators [15, 16].

Boßlau et al. (2021) have recently shown that the proportion of circulating CD8^+^ EMRA T-cells as well as plasma levels of KYN are associated with abdominal obesity and represent potential predictors of progressive glucose intolerance in the elderly [17]. These findings have been supported by the results of studies using the mouse model [18, 19]. Yi et al. (2019) transferred senescent CD8^+^ T-cells (CD44^+^CD153^+^) from mice fed a high-fat diet into young recipient mice, which subsequently exhibited worsened systemic glucose tolerance and decreased insulin sensitivity compared with control mice. In addition, the researchers demonstrated that senescent CD8^+^ T-cells (CD28^−^CD57^+^) are more numerous in human subjects with prediabetes and express a variety of proinflammatory cytokines and cytotoxic substances [18]. Laurans et al. (2018) showed that a high-fat diet in mice increases the expression of IDO in various tissues, thereby directing the metabolism of TRP towards KYN and its downstream products KA and quinolinic acid (QA). However, IDO knockout mice were protected from the obesogenic, inflammatory, and insulin resistance-inducing effects of the diet [19].

Given the high clinical and health economic relevance of metabolic diseases, it is of central interest to verify associations between CD8^+^ EMRA T-cells, KYN pathway metabolites, and insulin resistance in humans. Furthermore, knowledge of potent therapeutic regimens would be desirable to attenuate progressive CD8^+^ T-cell differentiation and rebalance the KYN pathway in the elderly, thereby potentially preventing the development of manifest type 2 diabetes mellitus.

Lifestyle modifications such as exercise or a healthy diet are becoming increasingly important in the prevention and treatment of age-associated diseases. Lifelong aerobic exercise has been shown to attenuate immune aging [20]. However, whether resuming exercise at older ages after years of inactivity also has beneficial effects on T-cell differentiation is much less clear. Niemiro et al. (2021) demonstrated that 12 weeks of moderate aerobic endurance training in a population of middle-aged/older women identified as being of high risk of developing breast cancer had a rejuvenating effect on the composition of T-cell subpopulations [21]. The extent to which such effects are transferable to healthy older individuals in the general population remains unclear. It is also unknown whether combined strength and endurance training produces similar effects compared to aerobic exercise programs. The impact of regular physical exercise on the KYN pathway has been analysed by only a few authors so far [22]. These have mainly examined psychiatric and neurodegenerative patient groups, and some of the work has methodological weaknesses so no clear conclusions can yet be drawn for healthy older individuals. Furthermore, no studies have examined potential synergistic effects of exercise and diet interventions on changes in T-cell differentiation and the KYN pathway.

In the present study the aim was to investigate the effects of a 12-week strength-endurance exercise intervention alone or combined with a nutritional intervention on T-cell differentiation, the KYN pathway metabolites and metabolic health. It was hypothesized that regular exercise has a ‘rejuvenating’ effect on the composition of the peripheral CD8^+^ T-cell pool and reduces the proportion of EMRA subsets. Furthermore, it was hypothesized that there is an influence on the KYN pathway and that a concomitant balanced diet causes additional adaptations compared to exercise training alone.

## Results

### Baseline characteristics

**Fig. 1:**
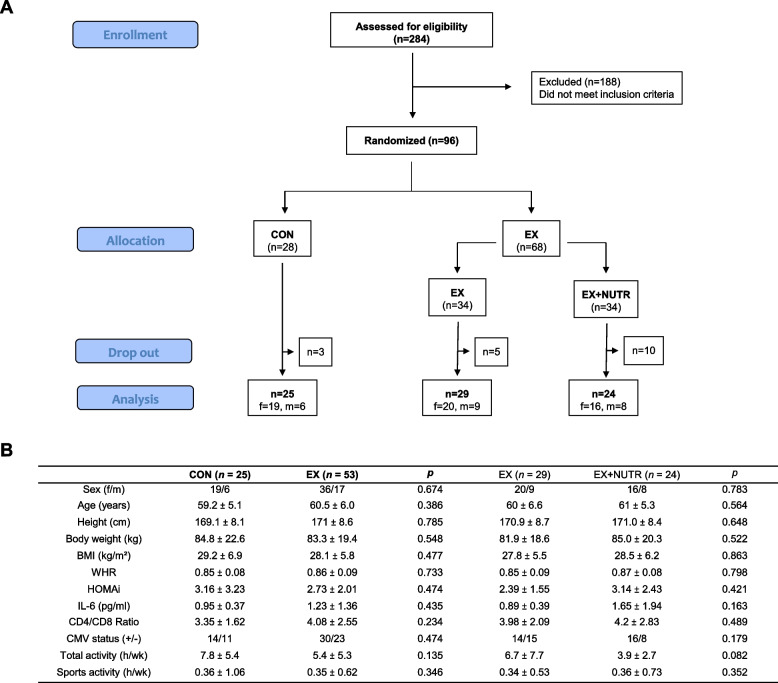
**A** Flow chart of all participants enrolled, randomized, allocated and analysed. **B** Baseline characteristics of participants, separated by study group. Values are given as mean ± SD. Distribution of sexes and CMV status between groups was analysed using Chi-square-test. All other group differences were assessed with Independent Samples T-Tests. Group differences between **CON** and **EX** (bold print) and between EX (normal print) and EX + NUTR were calculated separately and reported as p-values in each case (* *p* < .05; ** *p* < .01; *** *p* < .001). **CON** = control group; **EX** (bold print) = exercise group (with and without nutrition instructions); EX (normal print) = exercise group without nutrition instructions; EX + NUTR = exercise group with nutrition instructions; f = female, m = male; BMI = Body-Mass-Index; WHR = Waist-to-Hip-ratio; HOMAi = Homa-Index, CMV = cytomegalovirus; h/wk = hours per week

After screening for eligibility, 96 participants were enrolled in the study, of which 78 were included in the final analysis (Fig. 1A). The reasons for there being dropouts within the intervention groups were due to inadherence to the training and nutrition intervention, whereas in the control group, increased daily activity led to exclusion. A higher dropout rate was recorded in the EX + NUTR group compared to the other groups. Of all participants analysed, 70% were female and 30% were male, with a mean age of 59.8 ± 5.6 years. The study population can be classified as healthy but pre-obese, with a mean weight of 83.9 ± 20.3 kg, a BMI of 28.6 ± 5.8 kg/m^2^, and a WHR of 0.85 ± 0.09.

At baseline, the groups did not differ in age, body composition, HOMA-IR, IL-6 plasma concentration, CD4/CD8 ratio, CMV status, or activity level (Fig. 1B).

### Associations between age, BMI, HOMA-IR, peripheral CD8^+^ EMRA T-cells, plasma IL-6 concentration and metabolites of KYN pathway

**Fig. 2:**
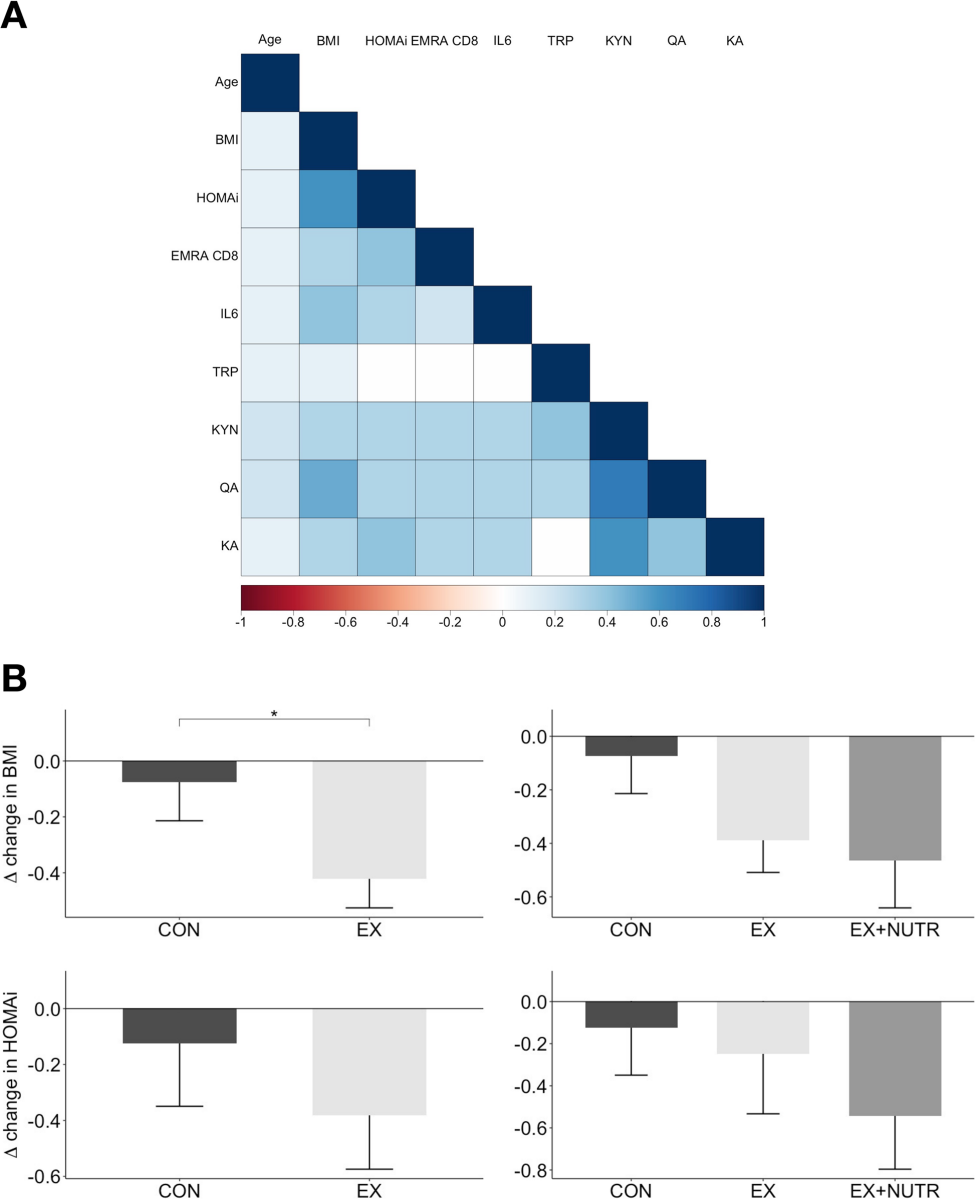
**A** Heat map illustrating baseline correlations. Pearson’s r values are depicted, where the values are given in the coloured scale bar. **B** Absolute change in BMI and HOMA-IR per study group over the twelve-week intervention period (Δ = t_12_—t_0_). *(*p* < .05) indicates a significant difference between groups, analysed by rmANCOVA. CON = control group; EX (two-group-comparison) = exercise group (with and without nutrition instructions); EX (three-group-comparison) = exercise group without nutrition instructions; EX + NUTR = exercise group with nutrition instructions; BMI = Body-Mass-Index, HOMAi = HOMA-Index; EMRA CD8 +  = Effector Memory re-expressing CD45RA CD8 + cells; TRP = tryptophane; KYN = kynurenine; QA = quinolinic acid; KA = kynurenic acid

Pearson's correlation of baseline values (t0) shows a significant positive relationship between BMI and HOMA-IR (Fig. 2A). Both BMI and HOMA-IR also correlate positively with IL-6 plasma concentration, proportions of CD8^+^ EMRA T-cells, as well as plasma levels of KYN and its metabolites QA and KA. The proportion of peripheral CD8^+^ EMRA T-cells shows a positive association with KYN, QA, and KA. IL-6 plasma concentration also correlates positively with KYN, QA, and KA. Pearson’s correlation of changes between the measurement time points (t12-t0) showed no significant correlations, except for the metabolites of the KYN pathway among themselves (data not shown).

### Manipulation check: Effects of the study intervention on BMI, HOMA-IR and maximum strength

The manipulation check revealed that BMI was significantly reduced after the intervention in the EX group when compared to the CON group (F(1, 75) = 4.97, *p* = 0.029). Within a three-group comparison, BMI decreased slightly more in the EX + NUTR group (exercise group with nutrition instructions) than in the EX ONLY group (exercise group without nutrition instructions), but without statistical significance (F(2, 74) = 2.49, *p* = 0.09).

For HOMA-IR, an analysis using rmANCOVA revealed no significant different changes between the CON and EX group (F(1, 75) = 1.45, *p* = 0.23). Moreover, there was no statistically significant difference within the three-group comparison (F(2, 74) = 0.72, *p* = 0.49). However, HOMA-IR tended to decrease the most in the EX + NUTR group (Fig. 2B). The raw BMI and HOMA-IR data and the line plots of the rmANCOVA analyses are included as Supplementary Material (Table S1, Figure S1).

Maximum strength increased significantly in the EX group for all exercises (Leg Press, Bench Press, Lat Pulldown, Back Extension, Rowing, Abdominal Flexion) at t12 compared to t0 (*p* < 0.001). No maximum strength data were collected in the CON group.

### Effects of the study intervention on proportions of peripheral CD8^+^ T-cell subsets

**Fig. 3:**
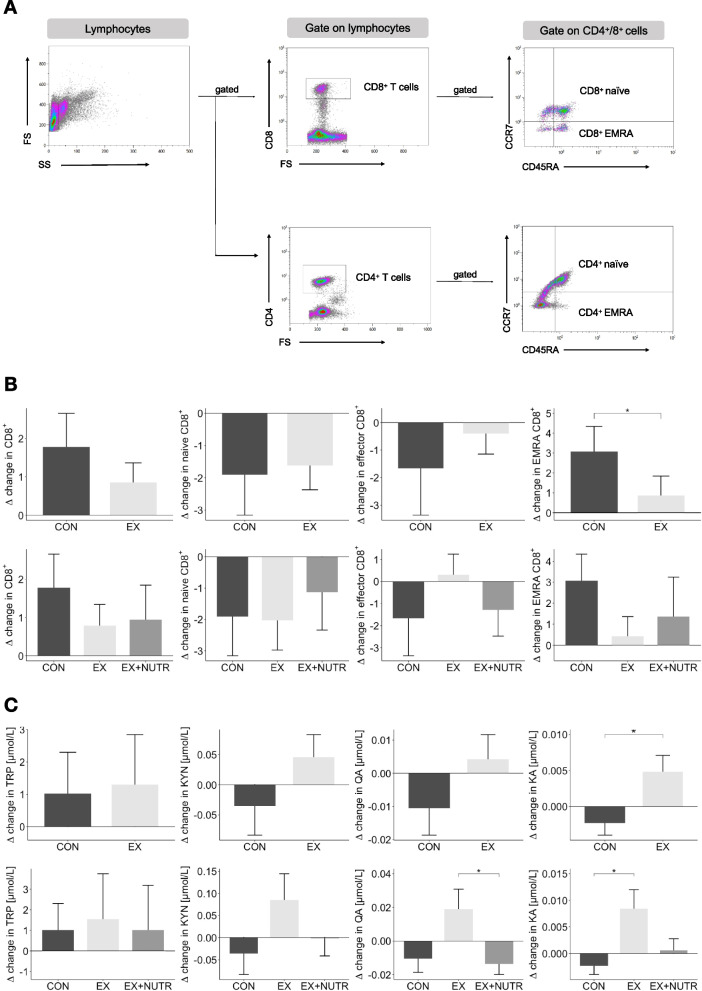
**A** Flow cytometric gating strategy for the analysis of T-cell subsets **B** Absolute changes in proportions of peripheral CD8^+^ T-cell subsets per study group over the twelve-week intervention period (Δ = t_12_—t_0_). *(*p* < .05) indicates a significant difference between groups, analysed using rmANCOVA. **C** Absolute changes of plasma metabolites of the KYN pathway in µmol/L (Δ = t_12_—t_0_). *(*p* < .05) indicates a significant difference between groups, analysed using rmANCOVA. CON = control group; EX (two-group-comparison) = exercise group (with and without nutrition instructions); EX (three-group-comparison) = exercise group without nutrition instructions; EX + NUTR = exercise group with nutrition instructions; EMRA CD8 +  = Effector Memory re-expressing CD45RA CD8 + cells; TRP = tryptophane, KYN = kynurenine; QA = quinolinic acid; KA = kynurenic acid

Between the CON and EX group, no significant different changes were observed for peripheral CD8^+^ T-cells (F(1, 60) = 0.36, *p* = 0.85) or the proportion of naïve (F(1, 63) = 0.13, *p* = 0.72), CM (F(1,63) = 0.79, *p* = 0.39) and EM subsets (F(1,63) = 3.13, *p* = 0.08). In contrast, the proportion of EMRA subsets changed statistically significantly between the CON and EX group (F(1, 63) = 5.68, *p* = 0.02), with a greater increase within the CON group (Fig. 3B). Within the three-group comparison, a statistically significant main effect was not seen for EMRA subsets (F(2, 62) = 2.83, *p* = 0.07). The raw data of the CD8^+^ T-cell subsets and the line plots of the rmANCOVA analyses are included as Supplementary Material (Table S2, Figure S1). An exploratory subgroup analysis, with CMV serostatus as an additional between-subject factor, did not reveal any significant ‘time x study group x CMV serostatus’ interaction effects on the proportions of the CD8^+^ T-cell subsets. However, a significant between-subjects effect was detected for the proportion of CD8^+^ EMRA T-cells, which depends on the CMV serostatus (Supplementary Table S3).

### Effects of the study intervention on plasma KYN pathway metabolites

For TRP and KYN, no statistically significant interaction effects were observed within the two-group or three-group comparison (Fig. 3C). For QA, the rmANCOVA analysis with the three-group comparison revealed a significant main effect (F(2, 72) = 4.38, *p* = 0.016) and a Bonferroni-corrected post-hoc analysis revealed a significant difference between the EX ONLY and EX + NUTR group, with an increase in the EX ONLY group and a decrease in the EX + NUTR group (*p* = 0.03, M_Diff_ = 0.033, 95%-CI[0.002, 0.064]). A significant intergroup effect was observed for KA concentrations, with greater values in the EX group and lower values in the CON group after the intervention (F(1, 73) = 4.79, *p* = 0.032). The three-group comparison also revealed a significant interaction effect (F(2, 72) = 3.76, *p* = 0.029) and a Bonferroni-corrected post-hoc analysis revealed a significant difference between the CON and EX ONLY group (*p* = 0.026, M_Diff_ = 0.005, 95%-CI[0.001, 0.02]). For the QA/KA-ratio, no statistically significant difference between groups was observed. However, a slight increase was seen in the CON group (+ 0.4), whereas it decreased in the EX ONLY (-1.0) and EX + NUTR group (-0.4). The raw data of the KYN pathway metabolites and the line plots of the rmANCOVA analyses are included as Supplementary Material (Table S4, Figure S1). An exploratory subgroup analysis with the CMV serostatus as an additional between-subject factor did not reveal any significant ‘time x study group x CMV serostatus’ interaction effect on the plasma levels of the KYN pathway metabolites. However, a significant between-subjects effect was detected for KYN, which depends on the CMV serostatus (Supplementary Table S5).

## Discussion

In this randomized controlled trial, it has been demonstrated that 12 weeks of combined strength/endurance training in the elderly had a positive effect on BMI and HOMA-IR, and attenuated the increase in the proportion of EMRA cells within the peripheral CD8^+^ T-cell compartment. Concomitant nutritional intervention showed no additional effect. Moreover, training affected the KYN metabolic pathway by increasing KA formation.

### Exercise training attenuates T-cell differentiation

The proportion of EMRA cells within the peripheral CD8^+^ T-cell compartment increased significantly in the CON group, while no changes were found in the EX group. Thus, combined strength/endurance training may attenuate the progressive increase in the CD8^+^ EMRA T-cell pool with age and seems to be a suitable approach for the delaying of T-cell differentiation. This is consistent with previous data: Vasconcelos et al. (2022) demonstrated in 108 postmenopausal women that both functional and combined strength-endurance training for 16 weeks (three times a week for 45 min each) reduced the proportion of EMRA cells in both the CD8^+^ and CD4^+^ T-cell compartments, compared with a control group [23]. In their study, the authors used the same surface markers as were used in this work for cell differentiation (CCR7, CD45RA) and the subjects also completed combined strength-endurance training, highlighting the reproducibility of the results of this work. Philippe et al. (2019) demonstrated an exercise-induced effect on T-cell differentiation in 16 older adults with prediabetes after an intervention period of only three weeks. In the study, subjects completed an aerobic exercise program three times weekly, running uphill (concentric) or downhill (eccentric) depending on the study group. The results showed that both protocols significantly decreased the percentage of CD4^+^ and CD8^+^ EMRA T-cells (CCR7-/CD45RO-), while increasing the proportion of central memory as well as naïve T-cells [24]. Dinh et al. (2019) examined the effects of six weeks (2–3 times per week) of exercise training on the number of senescence-prone CD8^+^ T-cells (CD57^+^) in 100 women aged ≥ 65 years [25]. They compared different exercise modalities: intensive strength training (80% of maximum repetition, 3 sets of 10 repetitions), strength endurance training (40% of maximum repetition, 2 sets of 30 repetitions), and a control condition (stretching). The authors found a significant decrease in the percentage and absolute count of senescence-prone CD8^+^ T cells in the strength endurance training group. The magnitude of change was also positively related to the number of training sessions. Interestingly, intense strength training did not affect the senescence-associated cell fraction. These results suggest that both modality and frequency of exercise may determine the training-induced effect on T-cell differentiation. This is consistent with Niemiro et al. (2021), who demonstrated divergent effects of 12-week endurance training (three times a week) on the numbers of CD4^+^ and CD8^+^ T-cell subsets in older women at risk of breast cancer depending on training intensity. Consequently, high-intensity training evoked more 'pro-senescent' (decrease in naïve CD4^+^ T-cells) and moderate-intensity training more 'anti-senescent' (increase in effector memory CD8^+^ T-cells) effects. However, training showed no significant effects on the CD8^+^ EMRA T-cell fraction, but the group size of the study was relatively small (n = 8), which should be considered critically [21]. Taken together, the current data suggest that CD8^+^ T-cell differentiation in the elderly is exercise-sensitive and can be influenced within a few weeks by both endurance and strength endurance training, depending on the training intensity and frequency. Further work comparing different training regimens is needed to make conclusive training recommendations. At present, only speculations can be made about the mechanisms of the training-induced adaptations in the CD8^+^ T-cell compartment.

It is conceivable that adrenergic stimuli and hemodynamic changes during repetitive exercise lead to a redistribution of highly differentiated T-cells into the blood [20], where they are exposed to pro-apoptotic factors such as reactive oxygen species [26, 27]. Since these cells in particular appear to be sensitive to these factors, they could be eliminated by repeated acute exercise [28] and subsequently shift the ratio of highly differentiated EMRA to naive CD8^+^ T-cells [29, 30]. This hypothesis is supported by Krüger et al. (2016) who showed that one session of high-intensity training (HIT) induced a higher increase of apoptosis in highly differentiated T-cells (CD28^−^CD57^+^) compared to a continuous exercise regimen [28]. Furthermore, from longitudinal data of Niemiro et al. (2021) it was revealed that changes in ˙VO2max were inversely related to changes in highly differentiated CD8^+^ EMRA T-cells after physical training. This has been confirmed by cross-sectional data [31], from which it has been shown that VO2max is inversely associated with the number of senescent CD8^+^ T-cells (KLRG1 + /CD57 + ; KLRG1 + /CD28-). Unfortunately, VO2max was not determined in the work of this study because the focus of the training intervention was on the strength endurance component. Instead, subjects in the EX group performed measurements of maximum strength before and after the intervention phase, but correlation analysis revealed no relationship between changes in maximum strength and changes in the proportion of peripheral CD8^+^ EMRA T-cells (data not shown). Physical training seems to reduce susceptibility to infection and could suppress reactivation of latent viruses [32, 33]. The increase in EMRA CD8^+^ T-cells in the control group might be due to the fact that they experienced more infections or a higher burden of latent viral infections than the exercise group. In particular, a persistent CMV infection is a known driver of T-cell differentiation and should be considered as a potential confounder in studies of physical activity and immunity [20]. However, in previous work examining the effect of exercise on CD8^+^ T-cell differentiation either the CMV status of the subjects at all [21, 23] was not recorded or it was not taken into account in the analyses with regard to an influence on the exercise-induced effects [24, 25]. This must be taken as a caveat and underscores the additional importance of the work of this study. In the work of this study, CMV serostatus was considered as a covariate in the rmANCOVA statistics. In addition, an exploratory subgroup analysis was performed with the CMV serostatus as an additional between-subject factor. This analysis did not reveal any significant ‘time x study group x CMV serostatus’ interaction effects. Thus, the CMV serostatus does not appear to influence the exercise-induced change on the proportions of peripheral CD8^+^ T-cell subsets. However, significant between-subjects effects were found for the proportion of CD8^+^ EMRA T-cells, thereby suggesting a general effect of latent CMV infection on these cells, which is consistent with a recent paper published by the author’s group [34].

Regarding dietary modification, no additional effects were observed on the distribution of peripheral CD8^+^ T-cell subsets. In general, to the best of the authors’ knowledge, there is only one study with aged nonhuman primates in which the effects of diet have been investigated on T-cell differentiation. In this it was shown that long-term caloric restriction delayed T-cell differentiation and reduced the production of inflammatory cytokines by memory T-cells [35]. In the EX + NUTR group of the work in this study, mean energy intake decreased by approximately 200 kcal per day after the intervention period, but this was not statistically different from the other groups, as described in a previously published paper of this same study [36]. Therefore, future human studies focusing more specifically on the effect of caloric restriction on T-cell differentiation would be interesting.

### Exercise training and dietary measures affect kynurenine pathway

Plasma concentration of KA increased significantly in the EX group compared with the CON group, whereas the concomitant dietary modification attenuated this effect. QA also showed an increase in the EX group, while the concomitant dietary modification had the opposite effect and decreased plasma concentration. Both KA and QA are derived from KYN, which in turn is the main product of tryptophan. KA is synthesized by KYN aminotransferases 1–4 (KAT 1–4) and has neuroprotective properties, whereas QA is closely associated with neuronal excitotoxicity and is produced by kynurenine-3-monooxygenase (KMO) [22]. To the best of the authors’ knowledge, the present work is the first in which a significant increase has been demonstrated in the plasma concentration of KA in response to a longitudinal training intervention. In comparable work only an increased gene expression of KAT isoenzymes in skeletal muscle was demonstrated [37, 38]. Moreover, Allison et al. (2019) observed a non-significant increase in KA and a non-significant decrease in QA in peripheral blood [38]. The reason why enhanced expression of KAT did not have a stronger effect on the concentration of KA in peripheral blood is unclear. Epigenetic influences may play a role here. In addition, rapid metabolism or excretion of the synthesized KYN metabolites are also speculated to be causative [39].

Within the work of this study, concomitant balanced dietary modification had a mitigating effect on the exercise-induced changes and resulted in reduced concentrations of KYN pathway metabolites. In particular, peripheral concentrations of QA were shown to have a significant reduction in the EX + NUTR group compared with the EX ONLY group. One possible explanation is that food rich in antioxidant compounds could counteract immune response and tryptophan breakdown by IDO. Jenny et al. (2011) showed that specific compounds like vitamin C and E, and also stilbene resveratrol and coffee flavonoids, were able to delay T-cell activation and IFN-γ production in vitro in mitogen-stimulated human peripheral blood mononuclear cells (PBMC) from healthy donors, followed by reduced activity of the downstream biochemical pathways like tryptophan breakdown by IDO-1 [40]. Since it has already been confirmed in preliminary work that the subjects in the EX + NUTR group consumed significantly more vegetables and fruit [36], this is a conceivable explanatory direction.

In summary, it is noticeable that the QA/KA ratio shifted slightly in favour of KA in the EX ONLY and EX + NUTR group, whereas it shifted in favour of QA in the CON group. In many neurodegenerative diseases, there is an imbalance between KA and QA in the CNS with an accumulation of QA and a decrease in KA [22]. In addition, the development of metabolic diseases has been associated with increased QA concentrations as well as decreased KA synthesis [41, 42]. Based on this, from the decreased QA/KA ratio in the intervention groups of this study it can be cautiously inferred that combined strength endurance training with or without a concomitant nutritional modification may have a moderate neuroprotective effect and could reduce the risk of developing type 2 diabetes mellitus in healthy elderly.

### Limitations

A substantially higher dropout rate occurred in the EX + NUTR group (n = 10) compared to the EX ONLY (n = 5) and CON (n = 3) group due to inadherence to the training and nutrition intervention. This should be taken into account when interpreting the data. However, the overall retention of subjects was good (> 80%), suggesting a high day-to-day suitability of the study intervention procedure. In this work, peripheral cell subsets were quantified by flow cytometry as percentages of the CD8^+^ T-cell fraction. Additional cell enumeration procedures were not performed, thus it was not possible to provide absolute cell concentrations. This is a significant limitation of the results, because it cannot be clearly demonstrated whether an increase or decrease in the proportion of a cell population resulted from an absolute change in that population or was due to a relative change in other cell proportions. Nevertheless, in the context of age-related advancing T-cell differentiation, the relative composition of the different CD8^+^ subsets is also considered as an important outcome. Moreover, in previous work it has been shown that the relative reduction in peripheral senescence-prone CD8^+^CD57^+^ T-cells (as a percentage of CD8^+^ T-cells) caused by strength-endurance training is related to an absolute decrease in concentration of these cells in the blood [25]. Thus, highly differentiated CD8^+^ T-cells such as those in the EMRA subpopulation appear to be exercise sensitive themselves. The parameters of the work in this study were determined only in peripheral blood, and no evidence of changes in other organs or tissue types could be obtained. For example, the quantitative change of the KYN pathway metabolites in cerebrospinal fluid due to physical training would have been interesting. In addition, gene expression analyses of the enzymes involved in the KYN signalling pathway would have been useful. In the case of T-cell subsets, it is not clear at present whether unequal redistribution to extravascular tissues may be responsible for the exercise-induced altered proportions in peripheral blood. Moreover, more sensitive measurement methods of T-cell differentiation exist (e.g., deep T-cell receptor beta sequencing), which should be included in future work.

## Conclusions

BMI and HOMA-IR are associated with the proportions of CD8^+^ EMRA T-cells as well as KYN pathway metabolites. 12 weeks of combined strength and endurance exercise were found to attenuate the age-related increase in the proportion of EMRA subsets within the peripheral CD8^+^ T-cell pool. In addition, exercise activated the KYN pathway and significantly increased serum level of KA. Thus, combined strength and endurance training seems to be a suitable approach to attenuate CD8^+^ T-cell differentiation in the elderly and to redirect the KYN pathway towards KA. However, the clinical relevance of these effects needs further investigation.

## Methods

### Study participants

Ninety six men and women were enrolled from the general population in Hannover, Germany, between August 2018 and March 2019. Recruitment was conducted using a wide distribution via advertisements in local newspapers and public notices. The inclusion and exclusion criteria for each participant were determined using a formalized questionnaire. Inclusion criteria for participation were age ≥ 50 and ≤ 70 years, no regular exercise training aside from the daily activities for at least 2 years, and stable body weight (± 5 kg) for at least 6 months. Exclusion criteria were defined as cardiovascular diseases (angina pectoris, myocardial infarction, stroke, peripheral arterial occlusive disease, heart failure, cardiac arrhythmia), type 1 and 2 diabetes, renal insufficiency and liver diseases, blood coagulation disorders, chronic gastrointestinal disorders (e.g., ulcers, Crohn's disease, pancreatic insufficiency), immunological diseases (e.g. autoimmune diseases), intake of immunosuppressive drugs or laxatives, intake of supplements containing n3-FAs, smoking, alcohol-, drug and/or medicine dependency, pregnancy or lactation, retraction of the consent by the subject, concurrent participation in another clinical study, and participation in a study in the last 30 days. Following the guidelines of the Declaration of Helsinki, written informed consent was obtained from all participants before participation in the study. Ethical approval was provided by the Ethics Commission of the Medical Chamber of Lower Saxony (Hannover, Germany) (Bo/07/2018, URL: https://www.drks.de/drks_web/setLocale_EN.do). This study is registered in the German Clinical Trial Register (DRKS00014322).

### Study design

The present single-centre, randomized, controlled trial in a parallel-group design is based on a cross-sectional study recently published by the authors’ group [17]. For this the baseline data (t0) from this publication were used and the subjects completed a 12-week intervention phase in the present study, with a new data collection after the intervention (t12).

After baseline analysis (t0), participants were randomly assigned by an independent researcher to one of two study groups based on covariates (in descending order: sex, BMI, and age): 1) control group (CON), 2) exercise group (EX). Subjects in the EX group were further randomly subdivided according to whether they also received a nutritional intervention (EX + NUTR) or not (EX ONLY). The CON group served as a control group, and participants in this group were asked to maintain their usual level of diet and exercise throughout the 12-week study period. The EX group was instructed to perform exercise training twice per week. Participants in the EX ONLY group were asked to maintain their usual diet. Participants in the EX + NUTR group were asked to balance their diet according to guidelines provided by the managers of the study.

### Exercise training

The exercise training was carried out in cooperating fitness centres after thorough instruction by a professional trainer. Each training session consisted of an initial warm-up followed by two rounds of a strength and endurance circuit.

The strength training consisted of six machine-based exercises (Leg Press, Bench Press, Lat Pulldown, Back Extension, Rowing, Abdominal Flexion) covering all major muscle groups. Each of these were performed for one minute. During the first training session, a maximum strength test was performed with three attempts for each exercise. The score of the best of the three trials was used to set the machines to 60% of the participants' maximum strength for the first two weeks of training. For the following six weeks, the load was increased by 10% and again by 5% for the last four weeks. In the last training session, the maximum strength test was repeated analogously to the initial measurement. Endurance training consisted of a four-minute session on cycle ergometers and cross-trainers with a perceived exertion corresponding to a score of 15/20 on the Borg scale [43]. Participants had 30 s of rest between each exercise. Including warm-up and rest periods, the training session could be completed in approximately one hour.

### Nutrition intervention

Participants in the EX + NUTR group received individualized nutritional counselling from a professional dietitian at the start. The dietary recommendations were based on the guidelines of the German Nutrition Society (DGE) [44], which are comparable to the recommendations of the World Health Organization (WHO) [45]. They generally included the following advice for the whole time period of the intervention: Daily consumption of 3 servings of vegetables and 2 servings of fruit, consumption of cereal products with an emphasis on whole grains, daily consumption of dairy products such as milk or cheese, limited meat consumption of 300–600 g per week, consumption of fish once or twice per week, and limited consumption of salt and especially sugar.

### Monitoring of food intake and physical activity

General compliance with the study instructions was monitored in all groups by fortnightly telephone calls. Compliance to the exercise intervention was additionally assessed using an exercise diary. Furthermore, participants' exercise data were recorded by the cooperating fitness centres and handed over to the study management. The amount of regular physical activity outside the intervention was recorded at baseline, after six weeks, and at the end of the study using the Freiburg Physical Activity Questionnaire [46]. Thus, altered physical activity habits of the participants could be excluded. Participants' dietary intake was monitored at baseline, after six weeks, and at the end of the study using 3-day dietary logs. The records were reviewed by dietitians for completeness, legibility, and plausibility. If necessary, ambiguities were clarified with participants. Energy and nutrient intakes were estimated using the PRODI6.4® software (Nutri-Science GmbH, Freiburg, Germany). In addition, consumption of specific food groups was assessed using the Food Frequency Questionnaire (FFQ) [47]. In the context of a previously published paper of from the same project, a significant increase in vegetables, fruit, and fish intake in the EX + NUTR group could be verified [36]. The subjects' activity levels outside the study intervention, as well as their dietary intake, can be seen in the Supplementary Material (Table S6 and S7).

### Anthropometric measurements

Waist and hip circumferences were measured using a measuring tape, when the participant was in a standing position. The Waist-to-hip-ratio (WHR) was calculated. Height was measured with a stadiometer in metres (seca GmbH & Co. KG, Hamburg, Germany) and body weight was measured on a digital scale to the nearest 0.1 kg (seca GmbH & Co. KG, Hamburg, Germany).The BMI was calculated by the ratio of weight to the squared height. All measurements were carried out by the same study personal.

### Blood sampling

Blood was collected in the morning after at least two and a half days of rest, and after overnight fasting (≥ 10 h) using serum, EDTA, and NaF glucose tubes (Sarstedt AG & Co. KG, Nümbrecht, Germany). Blood was either processed directly (analysis of T-cell subpopulations, blood glucose, insulin resistance) or stored at -80 °C in serum and plasma form for later analyses (analysis of KYN pathway metabolites, IL-6, CMV status).

### Analysis of glucose metabolism and insulin resistance

An analysis of markers of glucose metabolism was performed by a certified laboratory (Laborärztliche Arbeitsgemeinschaft für Diagnostik und Rationalisierung e.V., in Hannover, Germany). Fasting glucose was analysed photometrically (Beckman Coulter GmbH, Krefeld, Germany). HbA1c was analysed using high-performance liquid chromatography (HPLC) (Bio-Rad Laboratories GmbH, Feldkirchen, Germany). For the determination of insulin the electrochemiluminescence immunoassay method (ECLIA) using cobas 801e (Roche Diagnostics GmbH, Mannheim, Germany) was applied. Insulin sensitivity was evaluated using the homeostatic model assessment (HOMA): HOMA-IR = fasting insulin (µU/ml) x fasting blood glucose (mg/dl) / 405 [48].

### Analysis of IL-6 plasma concentration

The plasma level of IL-6 was determined using a human Magnetic Luminex Assay (Bio-Techne, Abingdon, Oxon, UK) and a Magpix Luminex instrument (Luminex Corp, Austin, Texas, US) according to the manufacturer’s instruction.

### Analysis of T-cell subpopulations

Peripheral Blood Mononuclear Cells (PBMCs) were isolated from fresh EDTA whole blood by ficoll density gradient centrifugation. After the PBMCs were washed, 1 × 10^6^ cells in 100 µl PBS were stained for 20 min in the dark with 5 µl of different fluorescence-coupled antibodies, respectively (BioLegend Inc., USA & ImmunoTools GmbH, Germany). The antibody cocktails were composed as follows for the analysis of CD8^+^ subtypes: anti-CD8-FITC (clone MEM-31), anti-CD197(CCR7)-PE (clone G043H7), and anti-CD45RA-PerCP (clone HI100). The percentages of naïve (CD45RA^+^/CCR7^+^), central memory (CD45RA^−^/CCR7^+^), effector memory (CD45RA^−^/CCR7^−^), and EMRA (CD45RA^+^/CCR7^−^) CD8^+^ T-cells were quantified by flow cytometer FC 500 using the CXP software (Beckman Coulter, USA). The gating strategy was implemented in accordance with Koch et al. (2008) [4] and is illustrated in Fig. 3A. In addition, the CD4^+^/CD8^+^ T cell ratio was determined.

### Analysis of tryptophan metabolites

Tryptophane (Trp) and its metabolites KYN, QA, and KA were measured via high-performance liquid chromatography (HPLC) coupled to a mass spectrometer (MS). The serum was stored in 50 µL aliquots at -80 °C until analysis. The analysis was performed on a Waters ACQUITY UPLC® system equipped with an ACQUITY UPLC® HSS T3 analytical column coupled to a Xevo® TQ-XS triple quadrupole mass spectrometer (Waters, Eschborn, Germany), as described elsewhere [49].

### Analysis of Cytomegalovirus (CMV) serostatus

Serum anti-CMV immunoglobulin G (IgG) antibodies were detected using a semi-quantitative sandwich enzyme-linked immunosorbent assay (ELISA-Viditest anti-CMV IgG, VIDIA, Czech Republic). The procedure followed the manufacturer’s instructions. End-point optical density was measured by the Emax Plus ELISA reader (Molecular Devices, USA).

### Statistical analysis

Distribution of all data was assessed using the Shapiro–Wilk-Test and Gaussian distribution. Outliers in all outcome measures, defined as z-scores > 3, were removed using winsorization. In total, 1.37% of all data points were winsorized with a maximum of three values per parameter (3.8%), as these values were considered to be supraphysiological due to technical errors. Descriptive statistics were generated for all measured variables and the results given as mean ± standard deviation. Baseline characteristics were tested for significant intergroup differences using the independent samples T-Test and the Chi-square test (for nominal data). The Pearson's correlation coefficient was calculated to show baseline associations between age, BMI, HOMA-IR, CD8^+^ EMRA T-cells, IL-6, and metabolites of KYN pathway among all subjects regardless of their study group. In addition, a Pearson's correlation analysis was performed with the intervention-induced changes (t12-t0) of the same parameters. Subsequently, a manipulation check was performed to determine whether the study intervention had the intended effect on participants. For this purpose, differences in the change in BMI and HOMA-IR between groups were analysed by using a separate baseline-adjusted repeated measures analysis of covariance (rmANCOVA). In addition, paired samples T-tests were performed to test for potential differences in maximum strength values of the intervention group between before and after the study intervention. The effect of the intervention on the proportions of CD8^+^ T-cell subsets and the KYN pathway was analysed using rmANCOVA. For this, age, BMI and CMV serostatus, as well-described confounding factors for peripheral T-cell characteristics, and plasma levels of KYN pathway metabolites [17, 34] were included as covariates in the model. An exploratory subgroup analysis was also performed with the CMV serostatus (pos./neg.) as an additional between-subject factor (rmANCOVA with study group x CMV serostatus x time interaction, including age and BMI as covariates). For all outcome measures, at first the EX versus CON group was compared. Additionally, a three-group comparison in which the EX group was subdivided into with/without nutrition intervention was performed. For the case of significant interaction effects within the three-group comparison, a Bonferroni-corrected post-hoc analysis was calculated in order to specify the intergroup differences. The level of significance was set at *p* < 0.05. Statistical analyses were conducted using IBM SPSS statistics version 28.

## Supplementary Information

**Additional file 1:** **Table S1.** BMI and HOMA-IR and their changes (Δ) between the start (t0) and end (t12) of the intervention. **Table S2.** Proportions of peripheral T cell subpopulations and their changes (Δ) between the start (t0) and end (t12) of the intervention. **Table S3.** Exploratory subgroup analysis regarding the influence of CMV serostatus (on exercise-induced effects) on the proportions of peripheral CD8^+^ T-cell subsets (rmANCOVA with study group x CMV serostatus x time interaction). **Table S4.** Plasma metabolites and ratios of KYN pathway and their changes (Δ) between the start (t0) and end (t12) of the intervention. **Table S5.** Exploratory subgroup analysis regarding the influence of CMV serostatus (on exercise-induced effects) on peripheral KYN pathway metabolites (rmANCOVA with study group x CMV serostatus x time interaction). **Table S6.** Questionnaire based physical activity levels outside the intervention of the participants at baseline (0), after six weeks (6) and 12 weeks after the intervention (12). **Table S7.** Dietary Intake of food groups at baseline (0), after six weeks (6) and at the end of the intervention (12). **Figure S1.** Trends in outcomes per study group over the 12-week intervention period.** A.** BMI and HOMA-IR. **B.** Proportions of peripheral CD8^+^ T-cell subsets. **C.** Plasma metabolites of the KYN pathway. # (*p* < .05) indicates a significant difference between groups, analysed using rmANCOVA. CON = control group, EX (two-group-comparison) = exercise group (with and without nutrition instructions), EX (three-group-comparison) = exercise group without nutrition instructions, EX+NUTR = exercise group with nutrition instructions, BMI = Body-Mass-Index, HOMAi = HOMA-Index, EMRA CD8+ = Effector Memory re-expressing CD45RA CD8+ cells, TRP = tryptophane, KYN = kynurenine, QA = quinolinic acid, KA = kynurenic acid.

## Data Availability

The datasets generated and/or analysed during the current study are not publicly available, but are available from the corresponding author on the basis of a reasonable request.
