# Supplementary material for: 12-week combined strength and endurance exercise attenuates CD8+ T-cell differentiation and affects the kynurenine pathway in the elderly: a randomized controlled trial
Source: Immun Ageing. 2023 May 9;20:19. doi: 10.1186/s12979-023-00347-7 (PMC10169370; doi:10.1186/s12979-023-00347-7)
Supplement: Supplementary file 1 — Additional file 1: Table S1. BMI and HOMA-IR and their changes (Δ) between the start (t0) and end (t12) of the intervention. Table S2. Proportions of peripheral T cell subpopulations and their changes (Δ) between the start (t0) and end (t12) of the intervention. Table S3. Exploratory subgroup analysis regarding the influence of CMV serostatus (on exercise-induced effects) on the proportions of peripheral CD8+ T-cell subsets (rmANCOVA with study group x CMV serostatus x time interaction). Table S4. Plasma metabolites and ratios of KYN pathway and their changes (Δ) between the start (t0) and end (t12) of the intervention. Table S5. Exploratory subgroup analysis regarding the influence of CMV serostatus (on exercise-induced effects) on peripheral KYN pathway metabolites (rmANCOVA with study group x CMV serostatus x time interaction). Table S6. Questionnaire based physical activity levels outside the intervention of the participants at baseline (0), after six weeks (6) and 12 weeks after the intervention (12). Table S7. Dietary Intake of food groups at baseline (0), after six weeks (6) and at the end of the intervention (12). Figure S1. Trends in outcomes per study group over the 12-week intervention period. A. BMI and HOMA-IR. B. Proportions of peripheral CD8+ T-cell subsets. C. Plasma metabolites of the KYN pathway. # (p < .05) indicates a significant difference between groups, analysed using rmANCOVA. CON = control group, EX (two-group-comparison) = exercise group (with and without nutrition instructions), EX (three-group-comparison) = exercise group without nutrition instructions, EX+NUTR = exercise group with nutrition instructions, BMI = Body-Mass-Index, HOMAi = HOMA-Index, EMRA CD8+ = Effector Memory re-expressing CD45RA CD8+ cells, TRP = tryptophane, KYN = kynurenine, QA = quinolinic acid, KA = kynurenic acid. [file 12979_2023_347_MOESM1_ESM.docx]

**Supplementary Materials**

**Table S1.** BMI and HOMA-IR and their changes (Δ) between the start (t0) and end (t12) of the intervention.

|  |  | | **CON** |  | **EX** |  | EX |  | EXDC |  |
| --- | --- | --- | --- | --- | --- | --- | --- | --- | --- | --- |
| **Parameter** | | **t** |  | **Δ** |  | **Δ** |  | Δ |  | Δ |
| BMI | | 0 | 29.18 ± 6.92 | -0.08 | 28.12 ± 5.82 | -0.43 | 27.79 ± 5.48 | -0.39 | 28.51 ± 6.22 | -0.46 |
|  |  | 12 | 29.11 ± 6.65 |  | 27.69 ± 5.61 |  | 27.40 ± 5.35 |  | 28.05 ± 6.13 |  |
| HOMA-IR | | 0 | 3.16 ± 3.23 | -0.1 | 2.73 ± 2.01 | -0.37 | 2.39 ± 1.55 | -0.25 | 3.14 ± 2.43 | -0.53 |
|  |  | 12 | 3.06 ± 3.01 |  | 2.36 ± 1.60 |  | 2.14 ± 1.01 |  | 2.62 ± 2.13 |  |

Values are given as mean ± SD and changes (Δ) between measurement time points are presented as mean differences. **CON** = control group, **EX** (bold print) = exercise group (with and without nutrition instructions), EX (normal print) = exercise group without nutrition instructions, EX+NUTR = exercise group with nutrition instructions, BMI = Body-Mass-Index, WHR = Waist-to-Hip-Ratio, HOMAi = Homa-Index.

|  |  | | **CON** |  | **EX** |  | EX |  | EXDC |  |
| --- | --- | --- | --- | --- | --- | --- | --- | --- | --- | --- |
| **Parameter** | | **t** |  | **Δ** |  | **Δ** |  | Δ |  | Δ |
| **CD8+ T cells** | | 0 | 23.9 ± 7.5 | 0.94 | 21.7 ± 8.0 | 0.92 | 21.1 ± 6.4 | 0.81 | 22.4 ± 9.7 | 1.09 |
|  |  | 12 | 25.7 ± 7.5 |  | 22.6 ± 7.9 |  | 21.9 ± 6.4 |  | 23.3 ± 9.6 |  |
| CD45RA+/ CCR7+ (naïve) | | 0 | 23.9 ± 15.9 | -1.94 | 27.4 ± 15.9 | -1.51 | 32.1 ± 18.7 | -1.69 | 21.5 ± 9.6 | -1.35 |
|  |  | 12 | 22.0 ± 14.2 |  | 25.9 ± 15.6 |  | 30.4 ± 18.6 |  | 20.8 ± 10.0 |  |
| CD45RA-/ CCR7+ (CM) | | 0 | 2.3 ± 2.1 | 0.45 | 3.5 ± 2.5 | 0.84 | 3.9 ± 3.1 | 0.61 | 3.1 ± 1.4 | 1.08 |
|  |  | 12 | 2.7 ± 2.2 |  | 4.3 ± 2.9 |  | 4.5 ± 3.4 |  | 4.2 ± 2.4 |  |
| CD45RA-/ CCR7- (EM) | | 0 | 39.7 ± 18.1 | -1.84 | 45.9 ± 13.3 | -0.91 | 41.3 ± 15.0 | 0.45 | 50.5 ± 9.4 | -1.87 |
|  |  | 12 | 37.9 ± 16.3 |  | 45.0 ± 13.2 |  | 41.7 ± 14.4 |  | 48.6 ± 11.1 |  |
| CD45RA+/ CCR7- (EMRA) | | 0 | 34.0 ± 20.6 | 3.33 | 23.9 ± 11.5 | 0.89 | 23.1 ± 12.0 | 0.30 | 24.8 ± 11.4 | 1.61 |
|  |  | 12 | 37.4 ± 19.7 |  | 24.8 ± 13.0 |  | 23.4 ± 13.3 |  | 26.4 ± 12.9 |  |
| **CD4+/CD8+ ratio** | | 0 | 3.4 ± 1,6 | -0.19 | 4.1 ± 2.6 | -0.32 | 4.0 ± 2.1 | -0.19 | 4.3 ± 2.8 | -0.58 |
|  |  | 12 | 3.0 ± 1,5 |  | 3.8 ± 1.7 |  | 3.8 ± 1.7 |  | 3.7 ± 1.8 |  |

**Table S2.** Proportions of peripheral T cell subpopulations and their changes (Δ) between the start (t0) and end (t12) of the intervention.

Values are given as mean ± SD and changes (Δ) between measurement time points are presented as mean differences. CD8+ T-cells are indicated as percentages of all CD3+ T-cells. Their subpopulations are indicated as percentages of CD8+ T-cells. **CON** = control group, **EX** (bold print) = exercise group (with and without nutrition instructions), EX (normal print) = exercise group without nutrition instructions, EX+NUTR = exercise group with nutrition instructions, CM = central memory, EM = effector memory, EMRA = effector memory re-expressing CD45RA.

**Table S3.** Exploratory subgroup analysis regarding the influence of CMV serostatus (on exercise-induced effects) on the proportions of peripheral CD8^+^ T-cell subsets (rmANCOVA with study group x CMV serostatus x time interaction).

|  |  | **Two-group comparison** | | | |  | **Three-group comparison** | | | | |
| --- | --- | --- | --- | --- | --- | --- | --- | --- | --- | --- | --- |
| **Proportion of CD8+ T-cells** |  | Between subjects effects  **CMV status** | | Within subjects effects  **Interaction time x group x CMV status** | |  | Between subjects effects  **CMV status** | | Within subjects effects  **Interaction time x group x CMV status** | | |
|  |  | F | *p* | F | *p* |  | F | *p* | F | *p* |  |
| Naïve  (CD45RA+/ CCR7+) |  | 2.071 | 0.155 | 0.010 | 0.920 |  | 0.406 | 0.527 | 0.015 | 0.986 |  |
| CM  (CD45RA-/ CCR7+) |  | 2.737 | 0.103 | 2.009 | 0.161 |  | 2.086 | 0.154 | 2.328 | 0.106 |  |
| EM  (CD45RA-/ CCR7-) |  | 0.304 | 0.583 | 0.686 | 0.411 |  | 0.994 | 0.323 | 0.372 | 0.691 |  |
| EMRA  (CD45RA+/ CCR7-) |  | 6.217 | **0.015** | 1.197 | 0.278 |  | 4.089 | **0.048** | 0.583 | 0.561 |  |

Data were analysed using two way repeated measure ANCOVA (rmANCOVA). Age and BMI were added as covariates in the model. Two-group comparison examined differences between the CON group and EX group. Three-group comparison examined differences between the CON, EX ONLY, and EX + NUTR group. The test statistic of the analysis of variance (F-value) and the level of significance (*p*-value) are given. Significant results (*p* < .05) are shown in bold. CON = control group, EX = exercise group with or without nutrition instructions, EX ONLY = exercise group without nutrition instructions, EX+NUTR = exercise group with nutrition instructions, CM = central memory cells, EM = effector memory cells, EMRA = effecor memory re-expressing CD45RA cells.

|  |  | | **CON** |  | **EX** |  | EX |  | EXDC |  |
| --- | --- | --- | --- | --- | --- | --- | --- | --- | --- | --- |
| **Parameter** | | **t** |  | **Δ** |  | **Δ** |  | Δ |  | Δ |
| Tryptophane (TRP) (µmol/L) | | 0 | 47.73 ± 12.48 | 1.02 | 49.96 ± 15.48 | 2.04 | 52.38 ± 15.3 | 2.90 | 47.03 ± 15.83 | 1.02 |
|  |  | 12 | 48.75 ± 12.1 |  | 52.0 ± 20.14 |  | 55.28 ± 22.19 |  | 48.05 ± 17.49 |  |
| Kynurenine (KYN) (µmol/L) | | 0 | 1.42 ± 0.4 | -0.04 | 1.34 ± 0.42 | 0.04 | 1.35 ± 0.36 | 0.09 | 1.32 ± 0.5 | 0 |
|  |  | 12 | 1.39 ± 0.32 |  | 1.38 ±0.38 |  | 1.43 ± 0.32 |  | 1.32 ± 0.45 |  |
| Kynurenic acid (KA) (µmol/L) | | 0 | 0.037 ± 0.011 | -0.002 | 0.034 ± 0.013 | 0.005 | 0.031 ± 0.009 | 0.009 | 0.037 ± 0.016 | -0.001 |
|  |  | 12 | 0.034 ± 0.009 |  | 0.039 ± 0.021 |  | 0.041 ± 0.023 |  | 0.036 ± 0.018 |  |
| Quinolinic acid (QA) (µmol/L) | | 0 | 0.43 ± 0.083 | -0.011 | 0.413 ± 0.072 | 0.006 | 0.412 ± 0.071 | 0.02 | 0.414 ± 0.075 | -0.014 |
|  |  | 12 | 0.419 ± 0.074 |  | 0.418 ± 0.077 |  | 0.432 ± 0.085 |  | 0.4 ± 0.066 |  |
| KYN/TRP | | 0 | 0.031 ± 0.008 | -0.002 | 0.028 ± 0.009 | 0.001 | 0.027 ± 0.01 | 0.001 | 0.03 ± 0.011 | -0.001 |
|  |  | 12 | 0.029 ± 0.006 |  | 0.029 ± 0.009 |  | 0.028 ± 0.01 |  | 0.029 ± 0.009 |  |
| KA/KYN | | 0 | 0.027 ± 0.008 | -0.002 | 0.026 ± 0.007 | 0.002 | 0.024 ± 0.006 | 0.004 | 0.028 ± 0.009 | 0 |
|  |  | 12 | 0.025 ± 0.005 |  | 0.028 ± 0.011 |  | 0.028 ± 0.013 |  | 0.028 ± 0.008 |  |
| QA/KYN | | 0 | 0.315 ± 0.066 | -0.007 | 0.329 ± 0.079 | -0.012 | 0.319 ± 0.066 | -0.01 | 0.341 ± 0.094 | -0.015 |
|  |  | 12 | 0.308 ± 0.043 |  | 0.317 ± 0.071 |  | 0.310 ± 0.062 |  | 0.325 ± 0.082 |  |
| QA/KA | | 0 | 12.4 ± 3.29 | 0.4 | 13.63 ± 4.73 | -0.72 | 14.04 ± 4.17 | -1 | 13.14 ± 5.48 | -0.38 |
|  |  | 12 | 12.8 ± 3.19 |  | 12.91 ± 5.32 |  | 13.03 ± 5.68 |  | 12.77 ± 5.1 |  |

**Table S4.** Plasma metabolites and ratios of KYN pathway and their changes (Δ) between the start (t0) and end (t12) of the intervention.

Values (in µmol/L) and ratios are given as mean ± SD and changes (Δ) between measurement time points are presented as mean differences. **CON** = control group, **EX** (bold print) = exercise group (with and without nutrition instructions), EX (normal print) = exercise group without nutrition instructions, EX+NUTR = exercise group with nutrition instructions.

**Table S5.** Exploratory subgroup analysis regarding the influence of CMV serostatus (on exercise-induced effects) on peripheral KYN pathway metabolites (rmANCOVA with study group x CMV serostatus x time interaction).

|  |  | **Two-group comparison** | | | |  | **Three-group comparison** | | | | |
| --- | --- | --- | --- | --- | --- | --- | --- | --- | --- | --- | --- |
| **Plasma level of KYN pathway metabolites** |  | Between subjects effects  **CMV status** | | Within subjects effects  **Interaction time x group x CMV status** | |  | Between subjects effects  **CMV status** | | Within subjects effects  **Interaction time x group x CMV status** | | |
|  |  | F | *p* | F | *p* |  | F | *p* | F | *p* |  |
| TRP |  | 0.878 | 0.352 | 0.003 | 0.957 |  | 0.664 | 0.418 | 0.005 | 0.995 |  |
| KYN |  | 4.040 | **0.048** | 0.014 | 0.976 |  | 4.649 | **0.035** | 0.694 | 0.503 |  |
| KA |  | 0.931 | 0.338 | 0.159 | 0.691 |  | 1.175 | 0.282 | 0.320 | 0.727 |  |
| QA |  | 1.152 | 0.287 | 1.515 | 0.222 |  | 1.827 | 0.181 | 1.941 | 0.151 |  |

Data were analysed using two way repeated measure ANCOVA (rmANCOVA). Age and BMI were added as covariates in the model. Two-group comparison examined differences between the CON and EX group. Three-group comparison examined differences between the CON, EX ONLY, and EX + NUTR group. The test statistic of the analysis of variance (F-value) and the level of significance (*p*-value) are given. Significant results (*p* < .05) are shown in bold. CON = control group, EX = exercise group with or without nutrition instructions, EX ONLY = exercise group without nutrition instructions, EX+NUTR = exercise group with nutrition instructions, TRP = tryptophane, KYN = kynurenine, KA = kynurenic acid, QA = quinolinic acid.

**Table S6.** Questionnaire based physical activity levels outside the intervention of the participants at baseline (0), after six weeks (6) and 12 weeks after the intervention (12).

| **Parameters** |  | **CON** | EX | EX+NUTR | ***p*** |
| --- | --- | --- | --- | --- | --- |
|  | **t** |  |  |  |  |
| Basal activity (hours/week) | 0 | 4.26 ± 3.81 | 4.17 ± 6.02 | 2.14 ± 1.86 | 0.257 |
|  | 6 | 4.84 ± 4.70 | 4.50 ± 3.76 | 2.95 ± 2.51 |  |
|  | 12 | 6.73 ± 10.0 | 6.80 ± 8.97 | 4.99 ± 4.31 |  |
| Leisure time activity (hours/week) | 0 | 3.04 ± 3.95 | 2.14 ± 2.74 | 1.16 ± 1.10 | 0.184 |
|  | 6 | 3.69 ± 4.66 | 2.39 ± 2.20 | 2.67 ± 2.00 |  |
|  | 12 | 4.36 ± 6.46 | 3.89 ± 5.59 | 2.65 ± 2.59 |  |
| Sport activity (hours/week) | 0 | 0.38 ± 1.04 | 0.33 ± 0.52 | 0.30 ± 0.69 | 0.468 |
|  | 6 | 0.40 ± 0.90 | 0.54 ± 1.21 | 0.32 ± 0.71 |  |
|  | 12 | 0.20 ± 0.47 | 0.50 ± 0.95 | 0.32 ± 0.70 |  |
| Total activity (hours/week) | 0 | 7.69 ± 5.34 | 6.63 ± 7.60 | 3.59 ± 2.56 | 0.151 |
|  | 6 | 8.93 ± 7.37 | 7.43 ± 5.66 | 5.94 ± 3.94 |  |
|  | 12 | 11.3 ± 14.5 | 11.2 ± 12.2 | 7.97 ± 6.02 |  |

Data shown as mean ± SD. Data were analysed using two way repeated measure ANOVA. P values represent time*intervention interaction. **CON** = control group, EX = exercise group without nutrition instructions, EX+NUTR = exercise group with nutrition instructions, t = time in weeks.

**Table S7.** Dietary Intake of food groups at baseline (0), after six weeks (6) and at the end of the intervention (12).

| **Parameters** |  | **CON** | EX | EX+NUTR | ***p*** |
| --- | --- | --- | --- | --- | --- |
|  | **t** |  |  |  |  |
| Fruit intake (portion/day) | 0 | 1.39 ± 1.06 | 1.85 ± 1.09 | 1.43 ± 0.98 | **0.006** |
|  | 6 | 1.27 ± 0.92 | 1.78 ± 1.42 | 1.96 ± 0.96 |  |
|  | 12 | 1.32 ± 0.78 | 1.83 ± 1.89 | 2.14 ± 1.26**‡** |  |
| Vegetable intake (portion/day) | 0 | 1.32 ± 1.87 | 1.10 ± 0.79 | 0.86 ± 0.34 | **0.001** |
|  | 6 | 0.95 ± 0.63 | 0.97 ± 0.70 | 1.17 ± 0.74 |  |
|  | 12 | 0.77 ± 0.48***** | 1.10 ± 0.97 | 1.49 ± 1.25**‡** |  |
| Meat intake (portion/day) | 0 | 1.63 ± 1.63 | 2.02 ± 2.04 | 1.33 ± 1.07 | 0.480 |
|  | 6 | 1.77 ± 2.31 | 1.57 ± 1.68 | 1.08 ± 0.77 |  |
|  | 12 | 1.51 ± 1.63 | 1.41 ± 1.45 | 1.06 ± 0.85 |  |
| Meat intake (g/week) | 0 | 621.9 ± 333.2 | 667.0 ± 494.9 | 528.81± 346.6 | 0.225 |
|  | 6 | 605.1 ± 384.2 | 485.3± 385.6 | 443.82± 256.3 |  |
|  | 12 | 536.4 ± 322.8 | 504.8 ± 327.1 | 418.1± 258.0 |  |
| Cereal intake (portion/day) | 0 | 3.38 ± 1.97 | 3.38 ± 2.37 | 3.03 ± 1.77 | 0.521 |
|  | 6 | 2.80 ± 1.71 | 2.81 ± 1.56 | 2.67 ± 1.03 |  |
|  | 12 | 2.52 ± 1.06**†** | 2.73 ± 1.55 | 2.75 ± 1.26 |  |
| Milk intake (portion/day) | 0 | 2.35± 1.80 | 3.02± 1.82 | 3.22± 2.15 | 0.366 |
|  | 6 | 2.22± 1.62 | 2.51± 1.41 | 3.20± 1.07 |  |
|  | 12 | 2.08± 1.48 | 2.51± 1.56 | 3.71± 2.47 |  |
| Fish intake (portion/week) | 0 | 1.23± 1.10 | 1.49± 2.18 | 1.77± 1.53 | **0.010** |
|  | 6 | 1.28± 0.99 | 1.16± 1.00 | 2.51± 1.94 |  |
|  | 12 | 1.08± 0.72 | 1.23± 1.06 | 2.69± 1.71**‡** |  |

Data shown as mean ± SD. P values represent time*intervention interaction analysed with two way repeated measure ANOVA. In case of significance, asterisks indicate statistical differences within groups detected with post hoc Bonferroni (* *p* < 0.05; † *p* < 0.01; ‡ *p* < 0.001). **CON** = control group, EX = exercise group without nutrition instructions, EX+NUTR = exercise group with nutrition instructions, t = time in weeks.


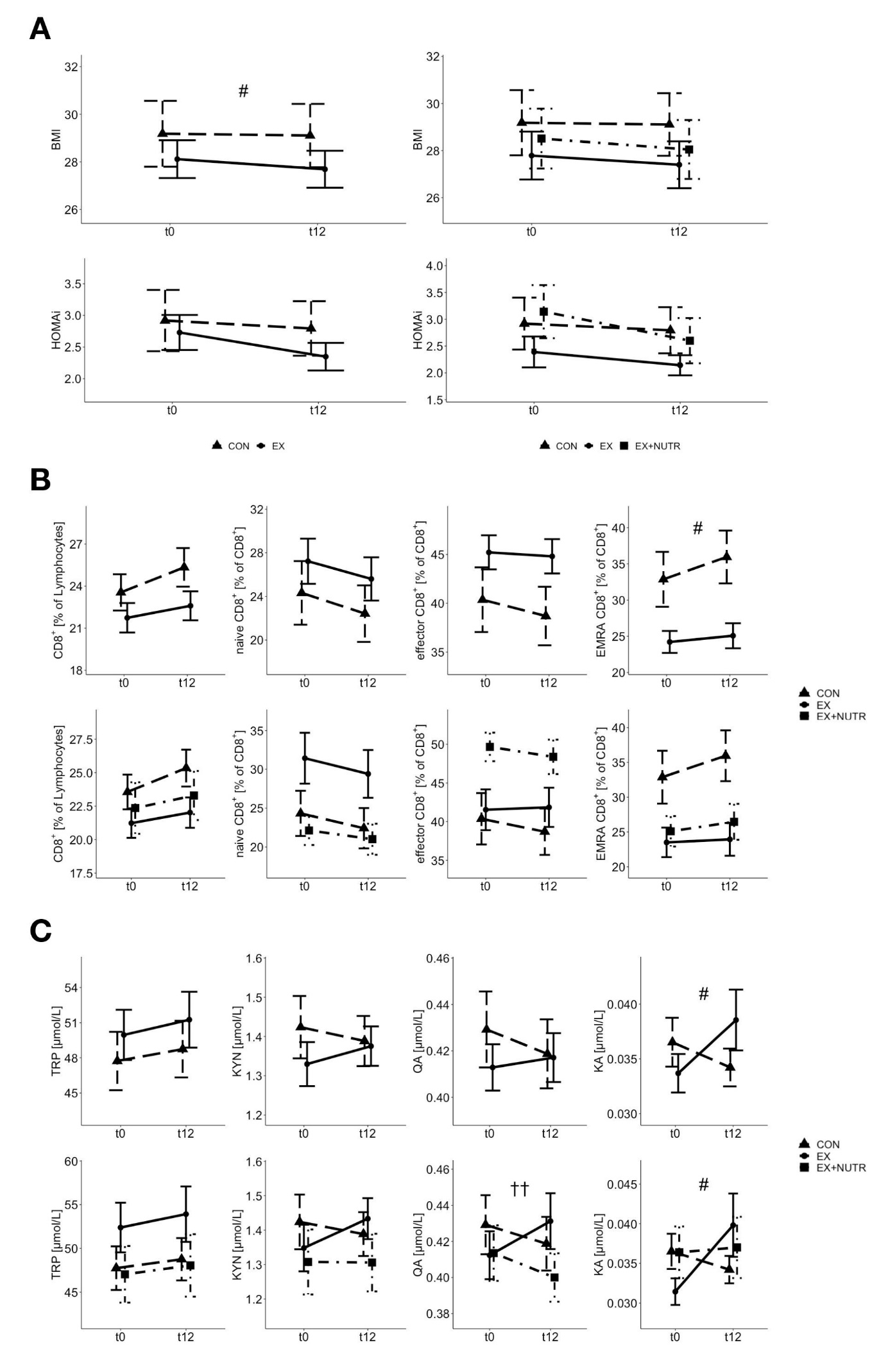


**Figure S1.** Trends in outcomes per study group over the 12-week intervention period. **A.** BMI and HOMA-IR. **B.** Proportions of peripheral CD8^+^ T-cell subsets. **C.** Plasma metabolites of the KYN pathway. # (*p* < .05) indicates a significant difference between groups, analysed using rmANCOVA. CON = control group, EX (two-group-comparison) = exercise group (with and without nutrition instructions), EX (three-group-comparison) = exercise group without nutrition instructions, EX+NUTR = exercise group with nutrition instructions, BMI = Body-Mass-Index, HOMAi = HOMA-Index, EMRA CD8+ = Effector Memory re-expressing CD45RA CD8+ cells, TRP = tryptophane, KYN = kynurenine, QA = quinolinic acid, KA = kynurenic acid.
